# Advanced Knowledge of Three Important Classes of Grape Phenolics: Anthocyanins, Stilbenes and Flavonols

**DOI:** 10.3390/ijms141019651

**Published:** 2013-09-27

**Authors:** Riccardo Flamini, Fulvio Mattivi, Mirko De Rosso, Panagiotis Arapitsas, Luigi Bavaresco

**Affiliations:** 1 Consiglio per la Ricerca e la Sperimentazione in Agricoltura-Centro di Ricerca per la Viticoltura (CRA-VIT), Viale XXVIII aprile, Conegliano 26-31015, TV, Italy; E-Mails: mirko.derosso@gmail.com (M.R.); luigi.bavaresco@entecra.it (L.B.); 2 IASMA Research and Innovation Centre, Fondazione Edmund Mach, San Michele all’Adige 38010, TN, Italy; E-Mails: fulvio.mattivi@fmach.it (F.M.); panagiotis.arapitsas@fmach.it (P.A.)

**Keywords:** grape, polyphenols, anthocyanins, stilbenes, flavonols

## Abstract

Grape is qualitatively and quantitatively very rich in polyphenols. In particular, anthocyanins, flavonols and stilbene derivatives play very important roles in plant metabolism, thanks to their peculiar characteristics. Anthocyanins are responsible for the color of red grapes and wines and confer organoleptic characteristics on the wine. They are used for chemotaxonomic studies and to evaluate the polyphenolic ripening stage of grape. They are natural colorants, have antioxidant, antimicrobial and anticarcinogenic activity, exert protective effects on the human cardiovascular system, and are used in the food and pharmaceutical industries. Stilbenes are vine phytoalexins present in grape berries and associated with the beneficial effects of drinking wine. The principal stilbene, resveratrol, is characterized by anticancer, antioxidant, anti-inflammatory and cardioprotective activity. Resveratrol dimers and oligomers also occur in grape, and are synthetized by the vine as active defenses against exogenous attack, or produced by extracellular enzymes released from pathogens in an attempt to eliminate undesirable toxic compounds. Flavonols are a ubiquitous class of flavonoids with photo-protection and copigmentation (together with anthocyanins) functions. The lack of expression of the enzyme flavonoid 3′,5′-hydroxylase in white grapes restricts the presence of these compounds to quercetin, kaempferol and isorhamnetin derivatives, whereas red grapes usually also contain myricetin, laricitrin and syringetin derivatives. In the last ten years, the technological development of analytical instrumentation, particularly mass spectrometry, has led to great improvements and further knowledge of the chemistry of these compounds. In this review, the biosynthesis and biological role of these grape polyphenols are briefly introduced, together with the latest knowledge of their chemistry.

## 1. Introduction

Grape contains a great number of classes of secondary metabolites: in particular, the composition of polyphenols is qualitatively and quantitatively very rich. The main polyphenols are anthocyanin, flavonol and stilbene derivatives, three classes of compounds displaying peculiar characteristics and which play important roles in plant metabolism [1].

Phenolic composition is highly affected by differences in grape varieties, environmental conditions and cultural practices. Berry phenolics contribute to wine quality and have beneficial effects on many aspects of human health. Due to their biological and organoleptic characteristics, anthocyanins, flavonols and stilbenes play a key role in wine quality, and grape extracts are used as sources of natural compounds in the pharmaceutical, food and nutraceutical industries [2]. As a consequence, studies are ongoing to improve knowledge of their chemistry, to better explain their roles in vine physiology, and to improve product characteristics.

In the last ten years, the technological development of analytical instrumentation has greatly improved and expanded knowledge of these compounds. In the 1990s, the development of Liquid Chromatography Mass Spectrometry (LC/MS) and Multiple Mass Spectrometry (MS/MS and MS^n^) supplied very useful techniques for studying polyphenol structures [3–5]. Many studies on anthocyanins in grape and wine have provided experimental evidence for structures which, until a few years ago, were only hypothesized, and we can now better understand the mechanisms in which they are involved in wine-making and wine aging [6]. A detailed qualitative and quantitative study of stilbene derivatives in grape by accurate MS was recently performed, and 18 stilbene derivatives, including monomers, glucoside derivatives, dimers (viniferins), trimers and tetramers were identified [7]. Study of grape flavonols by LC/MS has also shown the presence of isorhamnetin, laricitrin and syringetin derivatives, in addition to those of myricetin, quercetin and kaempferol which usually occur in wines [8].

In the present review, the biosynthesis and biological role of these classes of polyphenols in grape are briefly introduced, together with the latest knowledge on their chemistry.

## 2. Biosynthesis of Grape Polyphenols

### 2.1. Flavonoids

**Figure 1:**
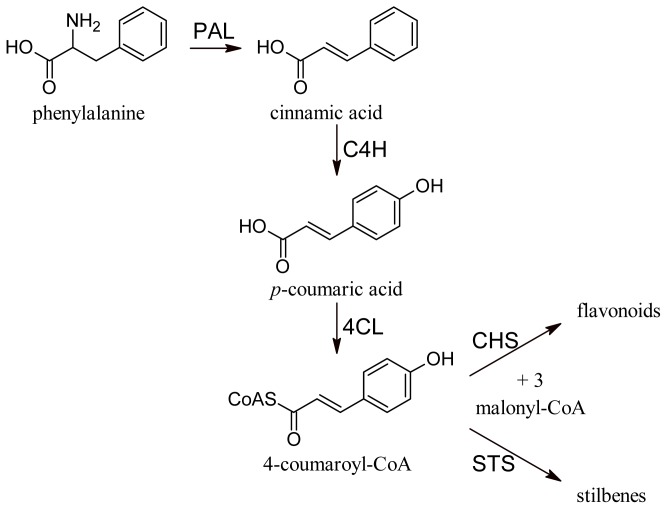
General phenylpropanoid pathway. PAL, phenylalanine ammonia lyase; C4H, cinnamate-4-hydroxylase; 4CL, 4-coumaroyl:CoA-ligase; CHS, chalcone synthase; STS, stilbene synthase.

**Figure 2:**
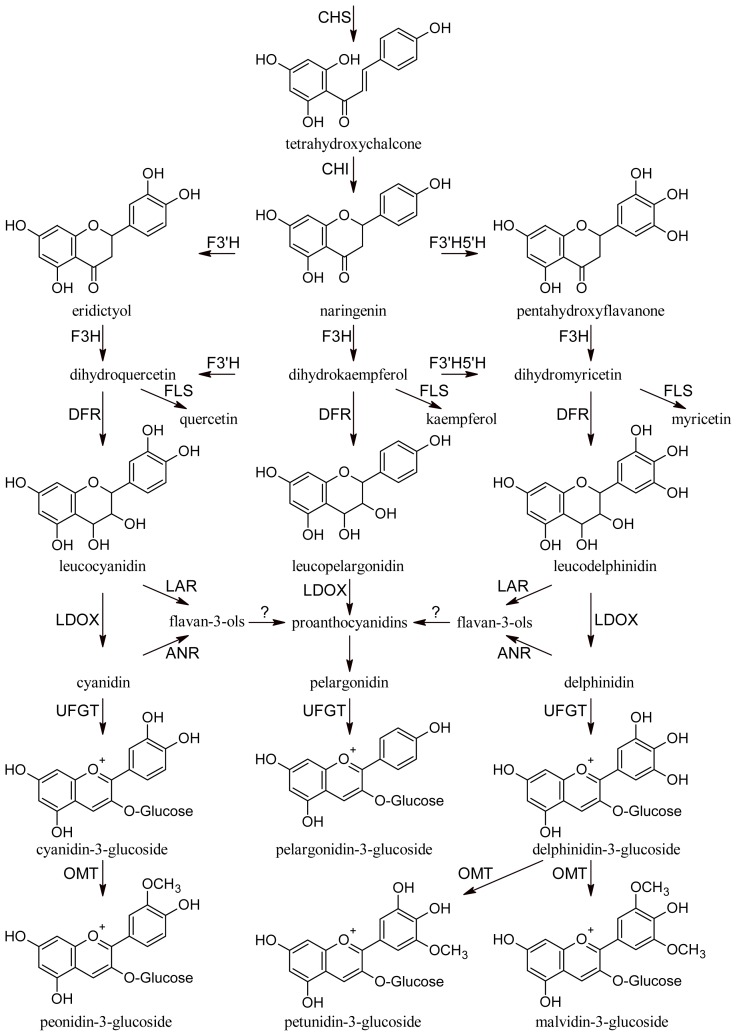
Flavonoid pathway. CHS, chalcone synthase; CHI, chalcone isomerase; F3′H, flavonoid-3′-hydroxylase; F3′5′H, flavonoid-3′,5′-hydroxylase; F3H, flavanone-3-hydroxylases; FLS, flavonol synthase; DFR, dihydroflavonol reductase; LAR, leucoanthocyanidin reductase; LDOX, leucoanthocyanidin dioxigenase; ANR, anthocyanidin reductase; UFGT, UDP-glucose:flavonoid 3-*O*-glucosyl transferase; OMT, *O*-methyltransferase.

All phenolic compounds are synthesized from the amino acid phenylalanine through the phenylpropanoid pathway [9]. Phenylalanine is in turn a product of the shikimate pathway, which links carbohydrate metabolism with the biosynthesis of aromatic amino acids and secondary metabolites. The general phenylpropanoid pathway is shown in Figure 1. Two main classes of compounds can be produced: flavonoids (by chalcone synthase) and stilbenes (by stilbene synthase). The flavonoid pathway leads to the synthesis of various classes of metabolites, such as flavonols, flavan-3-ols, proanthocyanidins and anthocyanins (Figure 2).

### 2.2. Anthocyanins and Flavonols

Flavonols are normally glycosylated at the C-3 position of the C ring. Anthocyanins are synthesized from anthocyanidins by glycosylation at the 3 and 5 positions of the C ring, are accumulated in berry skins and also in the flesh of some “teinturier” varieties, from veraison until full maturity, when synthesis stops. Anthocyanidin glycosides are generally more stable than the corresponding aglycones, as glycosylation induces intramolecular H-binding within the anthocyanin molecule [10]. Enzyme UDP-glucose:flavonoid 3-*O*-glucosyl transferase (UFGT) catalyses glucosylation of both anthocyanidins and flavonols, but its efficiency is much higher for the former [11]. Acylated anthocyanins are present in most red grapes, probably due to the presence of the enzyme anthocyanin acyl-transferase.

Anthocyanins are synthesized in the cytosol and delivered into the vacuole, where they are stored as colored coalescences called anthocyanic vacuolar inclusions. Vacuolar uptake may depend on mechanisms mediated by tonoplast transporters or based on vesicular trafficking. Transporter-mediated uptake may rely on two mechanisms. MATE-type proteins are localized to the tonoplast and function as vacuolar H^+^-dependent transporters of acylated anthocyanins [12]. ATP-binding cassette proteins are glutathione *S* conjugate pumps involved in the uptake of glycosylated flavonoids, independent of the presence of an H^+^ gradient. Glutathione S-transferases (GSTs) are thus expected to participate in vacuolar trafficking. They include a member of the *GST* gene family that has overlapping transcription patterns with anthocyanin accumulation [13,14]. Another putative anthocyanin carrier with high similarity to mammalian bilitranslocase has been isolated from grape berries [15]. Although evidence exists for a number of transporters, whether anthocyanins enter the vacuole as single molecules and then aggregate, or whether cytoplasmic vesicles containing coalesced anthocyanins interact with the tonoplast, remains unknown.

### 2.3. Stilbene Derivatives

Although flavonoids are found in all higher plants, only a few species produce stilbenes. Stilbene synthase (STS) and chalcone synthase (CHS) are closely related enzymes that specifically control flavonoid or stilbene biosynthesis. STS has evolved from CHS several times in higher plants and a few amino acid exchanges are sufficient to switch from chalcone to stilbene synthesis [15]. After its formation in the grapevine lineage, the *STS* gene has massively proliferated in the grapevine genome, mostly by local duplications, giving rise to a family of 43 members. To what extent each gene copy is redundant or whether any specialization has occurred is still unknown. In contrast, only three copies of CHS are present in the grapevine genome, with diversified patterns of expression in the grape berry [16]. Of other enzymatic steps which convert resveratrol into downstream derivatives, a resveratrol *O*-methyltransferase (ROMT) catalyses the conversion of resveratrol into the highly fungitoxic pterostilbene [17], and a glucosyltransferase can produce glucosides of *cis*- and *trans*-resveratrol and also has residual glucosyl activity on hydroxycinnamic acids and some flavonoids [18].

STS accepts as substrates 4-coumaroyl-CoA and 3 molecules of malonyl-CoA. Like CHS, STS carries out three reactions of condensation that produce resveratrol. In the STS reaction, the terminal carboxyl group is removed prior to closure of the A ring, which causes different ring-folding in resveratrol compared with tetrahydroxychalcone, the product of CHS. CHS and STS thus compete for the same substrates and control the entry points into the flavonoid and stilbene pathways, respectively.

### 2.4. Effects of Agrochemicals and Plant Activators on Grape Polyphenols

Biosynthesis of anthocyanin can be influenced by exogenous elicitors and different chemical compounds have been tested to increase their content in grape berries. Use of hormones, such as abscisic acid (ABA), jasmonate compounds, ethylene, salycilic acid, and non-hormone chemicals, such as ethanol and eutypine, were shown to promote the anthocyanin biosynthesis [19]. Recent studies showed also an increase of anthocyanin content in grapes treated with benzothiadiazole (BTH) and a BTH/methyl jasmonate mixture [20,21]. Also chitosan (a linear polysaccharide) and pectin-derived oligosaccharides have shown an increase the polyphenolic and anthocyanic content in grape [22,23].

Agrochemicals and plant activators act in the vine as elicitor also in the stilbene synthesis. The most used agrochemical is fosetyl-Al, a systemic fungicide active against Oomycetes fungi, like *Plasmopara viticola*, which induces the synthesis of *trans*-resveratrol and ɛ-viniferin in the leaves [24]. Plant activators include a large array of organisms, such as *Botrytis cinerea*, *Plasmopara viticola*, *Tricoderma viride*, *Erysiphe necator*, *Rhizopus stolonifer*, *Aspergillus carbonarius*, *Aspergillus japonicus*, *Laminarina* spp., *Bacillus* spp., and many products such as aluminium chloride, ozone, sucrose, dimethyl-β-cyclodextrin, MeJ, BTH, chitosan oligomers, salicylic acid, ABA, beta-amino-butyric acid (BABA), emodin, and treatments with UV rays [25–27].

While the influence of agrochemicals and plant activators on grape stilbenes, flavan-3-ols [28] and anthocyanins is a known issue due to SAR (systemic acquired resistance) and activators induce the expression of phenylpropanoid genes in grapevine [29], little is reported about their influence on flavonols. An induction of their synthesis, similar to that observed for anthocyanins, could be expected, given the closeness of the two biosynthetic pathways. In a “one vintage (2004)/one variety (Merlot)/one activator (BTH)” experiment, Iriti *et al*. revealed a very poor effect in the total flavonoids amount [28]. However, this study was not specific for flavonols. In a two-year study on treatment with BTH and methyl jasmonate (MeJ) conducted on Monastrell grape (2009–2010), an increase in flavonol concentration was observed [20] but the wide biological variability makes the results not completely consistent for the two years investigated. BTH-treated grapes had a higher flavonol concentration in both years (+17% and +56%), while the treatment with MeJ increased flavonols only in one year even if with a more pronounced effect (+131%). Differences between treated/untreated samples was more evident in the colder-humid year (2010), in which the conditions were more favorable for the development of pathogens, but no difference in the flavonol profile was observed. In any case, the influence of agrochemicals and plant activators in flavonoid synthesis is still an unclear argument in which further studies are needed.

## 3. Chemistry and Proprieties of Anthocyanins

In general, polyphenols are characterized by antioxidant activity, and *in vitro* studies have shown that they act as radical peroxyl scavengers in the formation of complexes with metals. Their ability to cross the intestinal wall of mammals (although some compounds show low bioavailability) and their cellular signaling activity has also been proven [1,30,31].

Anthocyanins are natural colorants present in the skin of red grapes that play a key role in the organoleptic characteristics of wines [32–34]. Anthocyanin accumulation also occurs in the pulp of the berries of a few “teinturier” varieties (which turn out to be slightly colored) with asymmetrical distribution within grape flesh and skins [35]. Environmental effects have a greater influence on the anthocyanin content in grape than their composition, which is more closely linked to variety. Anthocyanins have different biological functions in plant tissues, such as protection against solar exposure and UV radiation, pathogen attacks, oxidative damage and attack by free radicals; they are also capable of attracting animals for seed dispersal and of modulating signaling cascades [19]. Together with the other polyphenolics, anthocyanins have been studied to evaluate the ripening stage of grape [36], for chemotaxonomic purposes, and for their biological properties such as antioxidant, antimicrobial and anti-carcinogenic activity, and their protective effect on the cardiovascular system [37–41]. In addition, they represent an important source of natural colorants for the food, nutraceutical and pharmaceutical industries [42–44].

**Figure 3:**
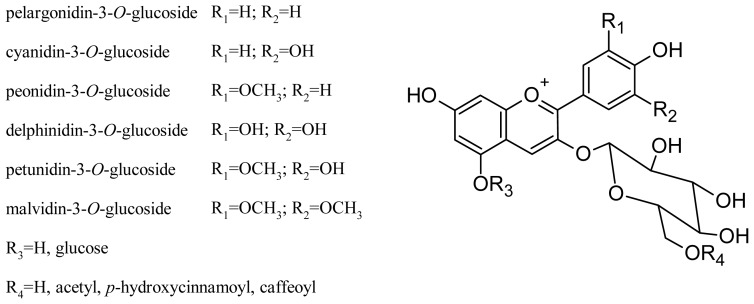
Structures of the principal *V. vinifera* grape anthocyanins.

Glycosylation at the C-3 and C-5 positions of the molecule affects the perceived color of the anthocyanin pigment: 3-glucoside derivatives are more intensely colored than 3,5-diglucosides, and acylation of glucose further increases the stability of the compound [45,46]. In general, *V. vinifera* grapes contain only 3-*O*-monoglucoside derivatives, due to two disruptive mutations in the anthocyanin 5-*O*-glucosyltransferase gene, which produces a nonfunctional form of the enzyme that normally performs 5-glycosylation in other grapevine species [47]. The main grape anthocyanins of these varieties are delphinidin, cyanidin, petunidin, peonidin and malvidin, present as monoglucoside, acetylmonoglucoside and *p*-coumaroylmonoglucoside derivatives. More recently, pelargonidin-3-*O*-glucoside and its acetyl and *p*-coumaroyl derivatives have been found [35,48,49]. The structures of the main *V. vinifera* anthocyanins are shown in Figure 3. Some anthocyanin oligomers have also been found in grape (Table 1) [50].

Each anthocyanin has a particular hue, ranging from red to blue. Delphinidin derivatives are associated with blueness and cyanidin derivatives are reddish. Methylation may affect color stability, as anthocyanin-*O*-methylation reduces the chemical reactivity of phenolic hydroxyl groups [51]. The combination of anthocyanin quantities and profiles contributes to the intensity and hue of the color of fruit and wines [52].

**Figure 4:**
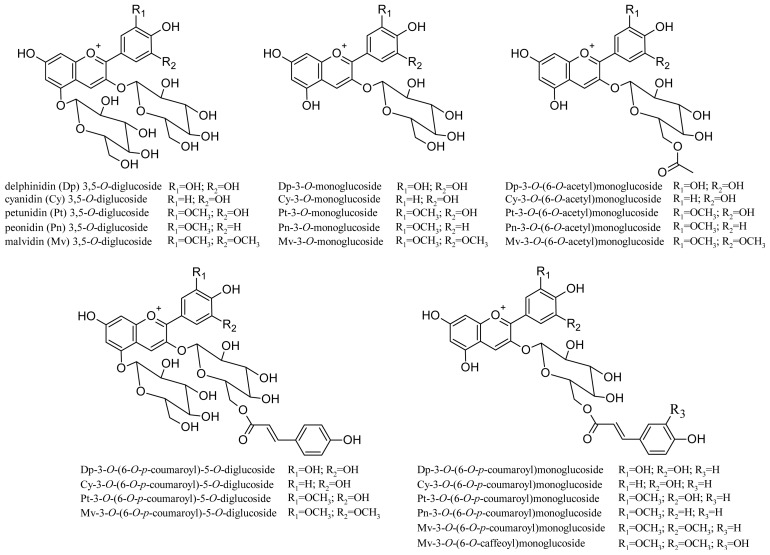
Structures of anthocyanins identified in 21 different hybrid red grape varieties.

Non-*V. vinifera* grapes (e.g., *V. riparia*, *V. labrusca*) also often contain 3,5-*O*-diglucoside anthocyanins. As they are practically absent in *V. vinifera* grapes, in several countries (e.g., the EU) some of these varieties cannot be used to produce wines (e.g., Isabelle, Clinton, and others [53]), and their presence in a wine is an indication of fraud. The anthocyanin composition of 21 hybrid red grape varieties produced by crossing *V. vinifera*, *V. riparia*, *V. labrusca*, *V. lincecumii* and *V. rupestris* species was recently studied [54]. LC/MS/MS analysis identified 24 anthocyanins, including 11 diglucoside derivatives (structures shown in Figure 4). As regards their content in grape, some varieties showed up to 5 g/Kg/grape of total anthocyanin (expressed as malvidin-3-*O*-glucoside). Table 2 lists other monomer anthocyanins identified in the extracts of some grape varieties.

## 4. Chemistry and Proprieties of Stilbene Derivatives

Stilbenes are phytoalexins naturally occurring in a few edible plants, and they also occur in grape. Their content increases from veraison to ripening, with significant differences among *V. vinifera* varieties [56]. These compounds, and in particular resveratrol, are among the main polyphenols associated with the beneficial effects of drinking wine. The main grape stilbenes are *cis-* and *trans*-resveratrol (3,5,4′-trihydroxystilbene), resveratrol-3-*O*-β-d-glucopyranoside (piceid), piceatannol (3,4,3′,5′-tetrahydroxy-*trans*-stilbene) and resveratrol dimers (viniferins) [57,58]. Isomeric and glycosylated forms of resveratrol and piceatannol, such as hopeaphenol, resveratroloside, resveratrol-4′-*O*-β-d-glucopyranoside, and resveratrol di- and tri-glucoside derivatives, have also been identified in trace amounts in grape [59]. Glycosylation of stilbenes is functional to storage, translocation, modulation of antifungal activity and protection from oxidative degradation. Glycosylated derivatives of resveratrol include both piceid and astringin (3′-hydroxy *trans*-piceid).

Several *in vitro* studies have shown that resveratrol has anti-cancer, anti-oxidant and anti-inflammatory activity, confers cardioprotection and inhibits platelet aggregation [60–66]. Pterostilbene (3,5-dimethoxy-4′-hydroxystilbene) is a dimethylated derivative of resveratrol with enhanced fungitoxic activity with respect to its precursor [59]. Piceatannol blocks LMP2A, a viral protein-tyrosine kinase implicated in leukemia, non-Hodgkin’s lymphoma and other diseases associated with the Epstein-Barr Virus (EBV) [67,68], and also acts on human melanoma cells [69]. Viniferins are produced through oxidation of the basic stilbene by 4-hydroxystilbene peroxidases. The most important are α*-*viniferin (a cyclic dehydrotrimer of resveratrol), β*-*viniferin (a cyclic dehydrotetramer of resveratrol), γ*-*viniferin (a more highly polymerized oligomer of resveratrol), δ*-*viniferin (an isomer of resveratrol dehydrodimer) and ɛ-viniferin (a cyclic dehydrodimer of resveratrol). Of these, ɛ*-*viniferin is the major stilbene synthesized in berries infected with *Botrytis cinerea* [70]. Synthesis of ɛ*-*viniferin and δ*-*viniferin can also be induced upon *Plasmopara viticola* infection or UV irradiation [71].

ɛ-Viniferins and ω-viniferins (E and Z isomers) and resveratrol trimers and tetramers (ampelopsin D, quadrangularin A, α-viniferin, E- and Z-miyabenol C, isohopeaphenol, ampelopsin H, vaticanol C-like) have been found in various parts of the plant, such as leaves, roots, clusters and stems, from oxidative coupling of resveratrol or resveratrol derivatives [72]. In *Vitis* grapevine canes, E-ampelopsin E, Eamurensin B, E-resveratroloside, E-3,5,4′-trihydroxystilbene 2-C-glucoside, Z-ampelopsin E, scirpusin A, E- and Z-vitisin B have also been found [73]. These compounds are formed by oligomerization of *trans*-resveratrol in grape tissue, a process induced as active defense by the plant against exogenous attack, or are produced by extracellular enzymes released from pathogens in an attempt to eliminate undesirable toxic compounds [74,75].

**Figure 5:**
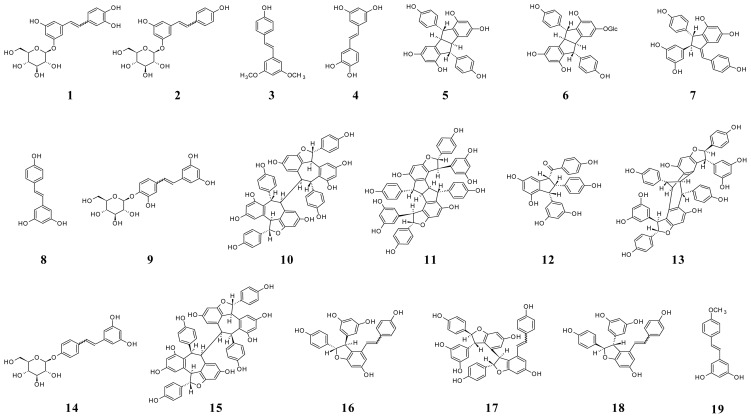
Structures of stilbenes in grape. (**1**) Z- and E-astringin; (**2**) Z- and E-piceid; (**3**) pterostilbene (3,5-dimethoxy-4′-hydroxystilbene); (**4**) piceatannol; (**5**) pallidol; (**6**) pallidol-3-*O*-glucoside; (**7**) parthenocissin A; (**8**) *trans*-resveratrol; (**9**) resveratroloside; (**10**) hopeaphenol; (**11**) ampelopsin H; (**12**) caraphenol B; (**13**) vaticanol C isomer; (**14**) resveratrol-4′-*O*-β-d-glucopyranoside; (**15**) isohopeaphenol; (**16**) E- and Z-ɛ-viniferin; (**17**) E- and Z-miyabenol C; (**18**) E- and Z-δ-viniferin; (**19**) *trans*-resveratrol-4′-methyl ether.

A study of stilbene derivatives in Raboso Piave and Primitivo grapes was recently performed by accurate and multiple (MS/MS) mass spectrometry assays [7]. A metabolomic approach by suspect screening analysis identified 18 stilbene compounds, some found in grape for the first time. Figure 5 shows the structures of stilbenes identified in grape.

## 5. Chemistry and Proprieties of Flavonols

Flavonols are secondary metabolites present in almost all higher plants. They are considered to act as UV- and photo-protectors because they absorb strongly at both UV-A and UV-B wavelengths. Flavonols also play an important role in wine copigmentation together with anthocyanins, are useful markers in grape taxonomy, and are considered bioactive grape/wine compounds of possible importance for human health and nutrition.

**Figure 6:**
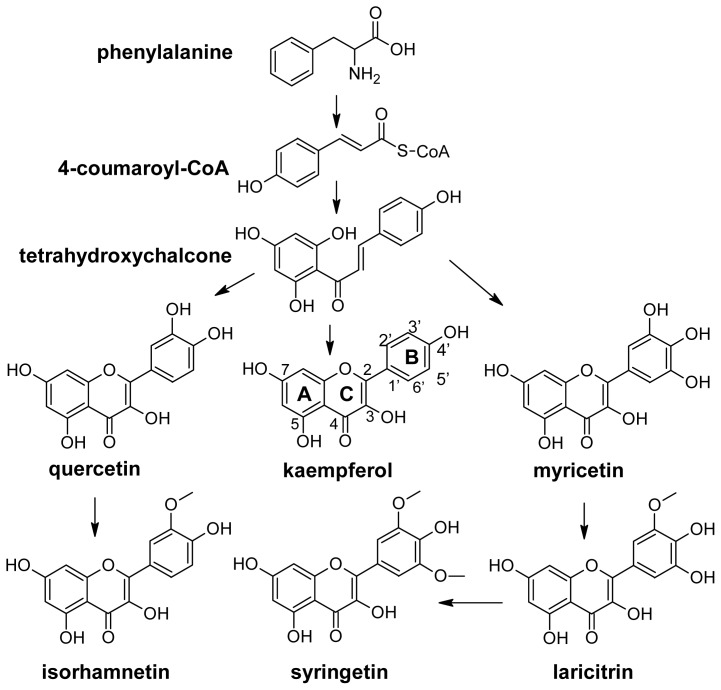
Flavonols pathway. The trihydroxylated flavonols myricetin, laricitrin and syringetin are lacking in the berries of white grapes.

The chemical structure of flavonols is closely related with their biosynthesis. Like all phenolic compounds, flavonols are products of the phenylpropanoid pathway, which converts phenylalanine into 4-coumaroyl-CoA and later into tetrahydroxychalcone (Figure 6). The biosynthesis of the various classes of flavonoids, which include flavonols, starts from this last metabolite. In general flavonols are C6-C3-C6 polyphenolic compounds in which two hydroxylated benzene rings, A and B, are joined by a three-carbon chain which is part of a heterocyclic C ring with a 3-hydroxyflavone backbone, and a double bond (Figure 6).

In detail, they differ in the number and type of substitution in the B ring. Kaempferol is monohydroxylated in position 3′; quercetin is dihydroxylated in positions 3′ and 4′; and myricetin is trihydroxylated in positions 3′, 4′ and 5′. Isorhamnetin is the methylated form of quercetin, and laricitrin and syringetin are the methylated forms of myricetin (Figure 6). All the above compounds normally occur in grape as glucosides, galactosides, rhamnosides, rutinosides and glucuronides [32,76–79]. Sugar is linked to position 3 of the flavonoid skeleton.

Flavonols are mainly located in the outer epidermis of the skin, since they act as UV-protecting agents. Their synthesis begins in the flower buttons, the highest concentrations being found a few weeks after veraison. It then stabilizes during early fruit development and decreases as the grape berries increase in size [80,81].

The total content and pattern of flavonols is highly variable across genotypes and can also be modulated to some extent by biotic and abiotic factors. Flavenol patterns are considered to be an important chemo-taxonomical parameter [8,32,41,82,83]. White and light red grape varieties synthesize mainly the mono- and di-substituted B-ring derivatives kaempferol, quercetin and isorhamnetin; red grapes also accumulate the tri-substitutes myricetin, laricitrin and syringetin [32,84]. Quercetin is thus the major flavonol of all white varieties such as Chardonnay, Riesling, Viogner and Sauvignon Blanc, in which it represents over 70% of total flavonols. Quercetin is also the major flavonol in some light red/rosé varieties such as Nebbiolo, Pinot Noir, Sangiovese and Gewuertztraminer, which also contain small percentages of myricetin (less than 20%). Conversely, myricetin is the major flavonol of most of the red varieties like Cabernet Sauvignon, Sagrantino and Teroldego [32]. Methylated derivatives are generally found in small quantities. All the flavonols detected in grape are glycosylated in position 3 of the B ring; no *C*-glycosylated compounds have been found. The sugar is usually glucose or glucuronic acid and galactose, rutinose or pentoses are found in smaller quantities.

The total amount of flavonols in grapes varies from 1 to 80 mg/Kg of fresh berry, the red cultivars often being richer than the white ones [32,41,78,84,85]. Some wild species of *Vitis* have been found to contain much higher amounts of flavonols than *Vitis vinifera* cultivars like *V. palmate* (124 mg/Kg) and *V. riparia* (111 mg/Kg) [82,83,86]. Another parameter which influences the amount of flavonols is the thickness of the berry skin, and thick-skinned grapes are reported to produce wines with higher amount of flavonols (e.g., Cabernet Sauvignon) than thin-skinned ones (e.g., Grenache) [87].

Agronomic and environmental factors also strongly affect mainly the amount and then the profile of flavonols in grape. In particular, flavonol biosynthesis in plant tissues is greatly influenced by sunlight. In general, it would normally be expected that grapes more exposed to daylight could enhance the biosynthetic pathway of all flavonoids [76,82,88–91]. Temperature appears to have a less significant influence [76,78]. Some recent studies have shown that high temperatures during maturation decrease the expression of genes related to flavonoid synthesis and favor anthocyanin biosynthesis. Day temperatures of 15–25 °C, falling to 10–20 °C at night, produced grapes with higher amounts of flavonols with respect to higher daytime temperatures (30–35 °C) [90]. Azuma *et al*. recently demonstrated that total flavonol amounts were higher for a daytime temperature of 15 °C under light treatment, with small variations in temperature, although in all cases the gene expression responsible for their biosynthesis was almost undetectable in bark-treated specimens [76]. UV radiation, particularly UV-B, activates flavonol biosynthesis, and any viticultural practice which favors exposing grapes to direct sunlight is expected to have a positive influence on their concentration in berries [76,77,80,91,92]. There is evidence that biosynthesis of flavonol is more strongly modulated by light than other berry metabolites. It has been shown that, although shading has a relatively modest effect on berry development and ripening, including accumulation of other flavonoids, it significantly decreases flavonol synthesis [92]. In ripe berries developed in opaque boxes, the presence of flavonols can be reduced by one order of magnitude [92].

## 6. Conclusions

Intimate structural knowledge of anthocyanins can be effectively applied in the chemotaxonomy of grapes, and is a powerful tool for evaluating polyphenolic ripening of grapes and developing new studies on biosynthetic pathways. It can also be used for enological purposes, to improve wine-making and wine aging methods. Study of non-*V. vinifera* grapes has shown that some varieties are qualitatively and quantitatively very rich in anthocyanins. Using these grape extracts in the natural colorant industry is potentially very interesting, partly due to the considerable contents of anthocyanin-acyl derivatives, which retain a more stable color in slightly acidic or neutral solutions [45].

The chemistry of stilbenes in grape compounds is very complex. These compounds act as phytoalexins, synthesized by plants in response to biotic and abiotic stress. They are also associated with the beneficial effects of drinking wine, and research focusing on developing agricultural and wine-making practices to increase their content in grapes and wines can be carried out. Lastly, producing stilbenes from sustainable sources is also desirable, due to the increased demand for them by the nutraceutical and pharmaceutical industries.

Among the flavonoids, flavonols are ubiquitous secondary metabolites found in all *Vitis* species. They are useful markers in *Vitis vinifera* chemotaxonomy, since their profiles in grapes are highly influenced by genetic factors at cultivar level. Flavonol profiles can thus be applied to classify both white and red varieties. In red varieties, their profiles are closely correlated with those of anthocyanins. The class of flavonols is also one of the most important grape flavonoids in terms of concentration, especially in white grapes. Their quantity in ripe berries is mainly influenced by environmental parameters, as a consequence of their photo-protective effect against excessive direct sunlight. Their presence has often been associated with the quality of grapes and wine, and the importance of these as two of the best sources of flavonols in the human diet should not be neglected.

The recent availability of advanced MS technologies has considerably improved our knowledge of the chemistry of grape and wine polyphenols. In the next few years, the momentum of applications of these analytical techniques in the study of grapes and wines will significant enhance further knowledge, leading to substantial benefits in the fields in which these polyphenolic compounds are of interest.

## Figures and Tables

**Table 1:** Oligomeric anthocyanins identified by liquid chromatography-mass spectrometry (LC/MS) analysis in Shiraz grape skins. F, fragment ion; M, molecular ion; Dp, delphinidin; Cy, cyanidin; Pt, petunidin; Pn, peonidin; Mv, malvidin; G, glucose; pCG, *p*-coumaroyl glucoside [50].

*m/z*	Assignment
287(F)	Cy
301(F)	Pn
303(F)	Dp
317(F)	Pt
331(F)	Mv
449(M)	Cy + G
463(M)	Pn + G
465(M)	Dp + G
479(M)	Pt + G
493(M)	Mv + G
617(F)	MvCy
631(F)	MvPn
633(F)	MvDp
647(F)	MvPt
661(F)	MvMv
779(F)	MvCy + G
793(F)	MvPn + G
795(F)	MvDp + G
809(F)	MvPt + G
823(F)	MvMv + G
941(M)	MvCy + 2G
955(M)	MvPn + 2G
957(M)	MvDp + 2G
971(M)	MvPt + 2G
985(M)	MvMv + 2G
1087(M)	MvCy + G·pCG
1101(M)	MvPn + G·pCG
1103(M)	MvDp + G·pCG
1117(M)	MvPt + G·pCG
1131(M)	MvMv + G·pCG
1271(F)	MvMvCy + 2G
1285(F)	MvMvPn + 2G
1287(F)	MvMvDp + 2G
1301(F)	MvMvPt + 2G
1315(F)	MvMvMv + 2G
1417(F)	MvMvCy + G·pCG
1431(F)	MvMvPn + G·pCG
1433(F)	MvMvDp + G·pCG
1433(M)	MvMvCy + 3G
1447(F)	MvMvPt + G·pCG
1447(M)	MvMvPn + 3G
1449(M)	MvMvDp + 3G
1461(F)	MvMvMv + G·pCG
1463(M)	MvMvPt + 3G
1477(M)	MvMvMv + 3G
1579(M)	MvMvCy + 2G·pCG
1593(M)	MvMvPn + 2G·pCG
1595(M)	MvMvDp + 2G·pCG
1609(M)	MvMvPt + 2G·pCG
1623(M)	MvMvMv + 2G·pCG

**Table 2:** Monomer anthocyanins identified in skin extract or juice of different grape cultivars [48,55]. Pg, pelargonidin; Dp, delphinidin; Cy, cyanidin; Pt, petunidin; Pn, peonidin; Mv, malvidin.

Anthocyanin	*m/z* (M^+^)	Cultivar
Cy-3-*O*-pentoside	419	Casavecchia
Pg-3-*O*-glucoside	433	Concord, Salvador, Rubired
Cy-3-*O*-(6-*O*-acetyl)pentoside	461	Casavecchia
Cy-3-*O*-(6-*O*-p-coumaryl)pentoside	565	Casavecchia
Dp-3-*O*-glucoside-pyruvic acid	533	Isabelle
Dp-3-*O*-(6-*O*-p-coumaryl)glucoside-pyruvic acid	679	Isabelle
Pn-3-*O*-glucoside-acetaldehyde	487	Isabelle, Pallagrello
Mv-3-*O*-glucoside-acetaldehyde	517	Isabelle
Pt-3-*O*-(6-caffeoyl)-5-*O*-diglucoside	803	Isabelle, Casavecchia
Dp-3-*O*-(6-acetyl)-5-*O*-diglucoside	669	Isabelle
Dp-3-*O*-(6-feruloyl)-5-*O*-diglucoside	803	Isabelle, Casavecchia
Pn-3-*O*-(6-O-*p*-coumaryl)-5-*O*-diglucoside	771	Concord, Salvador, Isabelle, Casavecchia
